# Risk analysis of microorganisms in common food spices

**DOI:** 10.3389/fmicb.2025.1549719

**Published:** 2025-08-25

**Authors:** Xiaoyang Tang

**Affiliations:** Shanghai Kangshi Food Science and Technology Co., Ltd., Shanghai, China

**Keywords:** food spices, microbial contamination, microbial input datasets, risk analysis, food safety

## Abstract

Spices play a ubiquitous role in the food industry, imparting distinctive flavors and exhibiting antibacterial properties. Nonetheless, microbial contamination and residues present persistent challenges to food safety within this sector. The systematic tracking of microbial input datasets in spices holds significant guiding implications and economic value for microbial control and risk assessment in the food industry. Furthermore, it serves as a cornerstone for developing early safety warning systems and precise prevention and control frameworks in spice processing. This paper, following a structured literature review methodology, conducts an in-depth analysis of microbial (bacterial and fungal) contamination across 41 commonly utilized spices. It elucidates the exposure risks associated with significant pathogenic and spoilage microorganisms prevalent in spices, categorizes the risk levels pertaining to spices and their corresponding microbial pollution profiles, and delineates an early warning framework for microbial contamination in spices. These findings furnish crucial insights for formulating control strategies targeting microorganisms in the spice sector and identifying key microbial control points within food processing operations.

**Systematic review registration:**

https://myfrontiers.frontiersin.org/projects/submission/1549719.

## 1 Introduction

Spices in food refer to substances added to food products to produce, modify, or enhance their flavor, as defined by GB 29938-2020. Natural spices for culinary purposes encompass a range of categories, including natural spices, strong aromatic spices, pungent spices, and delicate flavor spices, totaling 77 types as classified by GB/T 21725-2017. These spices find widespread application across the global food industry, utilized for seasoning, enhancing the taste of meat products, enriching convenience foods, preparing various dishes, and preserving food (Liu et al., 2022; Wang and Wang, 2021). The global spice market continues to witness expansion, propelled by escalating consumer demand. Notably, India emerged as the largest spice producer, with an annual production growth rate of 5.3% since 2014, reaching 2,176,908 tons in 2020. Turkey, in 2020, exported spices valued at $175.5 million, while Bangladesh produced 167,205 tons of spices. China, ranking fourth globally, produced 104,766 tons of spices, with an anticipated annual growth rate of 8.85%. Indonesia followed closely, producing 96,849 tons of spices (Yadav, 2024).

Numerous spices, including cloves, oregano, thyme, cinnamon, and cumin, contain a diverse array of bioactive compounds known for their pronounced inhibitory effects against pathogenic and spoilage microorganisms commonly found in food, such as *Staphylococcus aureus*, *Vibrio parahemolyticus*, *Salmonella typhimurium*, *Bacillus subtilis*, and *Bacillus fluorescens* (Liu et al., 2017; Zeng et al., 2015; Zhou et al., 2015; Zhang and Guo, 2011). These compounds play a pivotal role in enhancing food safety and quality while extending the shelf life of food products. Nevertheless, spices can serve as potential carriers of various foodborne pathogens and other contaminants, such as heavy metals and pesticide residues, which may be introduced during the spice addition process in diverse food preparations (Ming and Liang, 2020; Yan et al., 2022).

The microbiome associated with spices constitutes a complex assemblage of microorganisms that colonize the surface and internal tissues of spices. This microbial community undergoes enrichment during cultivation, harvesting, preservation, and initial processing stages. While the majority of microorganisms within this microbiome are innocuous, certain attached microbes may harbor pathogenic potential and contribute to post-harvest spoilage. Notably, between 1973 and 2010, microbial contamination of spices led to 14 documented outbreaks of foodborne illnesses. Among these, three reported incidents in the United States linked to *Salmonella* serotypes contamination in spices resulted in approximately 13,400 cases of foodborne infections, imposing a substantial burden on food safety efforts. Consequently, this underscores significant challenges in devising effective control strategies to mitigate the proliferation of harmful microorganisms during spice processing (Chen and Zhang, 2011; Ming and Liang, 2020; Van Doren et al., 2013).

The comprehensive comprehension of the microbiome associated with food spices holds paramount importance for the targeted prevention and management of microbial populations during food processing operations. This research endeavors to assemble datasets encompassing pertinent pathogenic and spoilage bacteria prevalent in spices via meta-analytical techniques. Through systematic analysis, the study elucidates the inherent risks posed by these pathogenic and spoilage bacteria within spices. Furthermore, it delineates the exposure risks and corresponding risk levels associated with spices and their associated harmful microorganisms. Such insights furnish a foundational framework for the establishment of early warning systems pertaining to food safety within the spice processing sector of the food industry. Concurrently, these findings serve as a pivotal cornerstone for the development of robust prevention and control strategies aimed at curtailing the proliferation of deleterious microorganisms.

## 2 Materials and methods

### 2.1 Source of the data and search strategy

Data for this bibliometric analysis were obtained from The Web of Science Core Collection, FSTA^®^-the food science resource [including the Social Sciences Citation Index (SSCI) and the Science Citation Index-Expanded (SCI-E)] and China National Knowledge Infrastructure (CNKI), accessed on 28 March 2024. The terms and keywords used for data retrieval include {spice, seasoning, cumin, Sichuan pepper, chili pepper, ginger, black pepper, and white pepper, microorganisms, microbe, bacteria, fungi, foodborne pathogen, etc.}.

### 2.2 Statistical methods

After selection by database collection criteria, a targeted sur-vey and tabular summary of key microbial contaminants was performed on a dataset that included 41 spice species. To further evaluate the potential exposure risk of spices to pathogenic microorganisms, spoilage microorganisms, and their combination, a thorough analysis of pertinent data was conducted. Data were analyzed using Excel, the Chiplot online analysis webpage,^1^ and GraphPad (version 10), and a matrix of microbial contamination of spices was plotted. This study undertook a comprehensive assessment of factors including the risk of exposure to microorganisms, prevalent processing methods, consumption patterns, and microbial characteristics. Subsequently, risk levels pertaining to spices and their associated microorganisms were determined, scored, and initially categorized.

## 3 Results and discussions

### 3.1 Microbial contamination status of food spices

**Figure 1:**
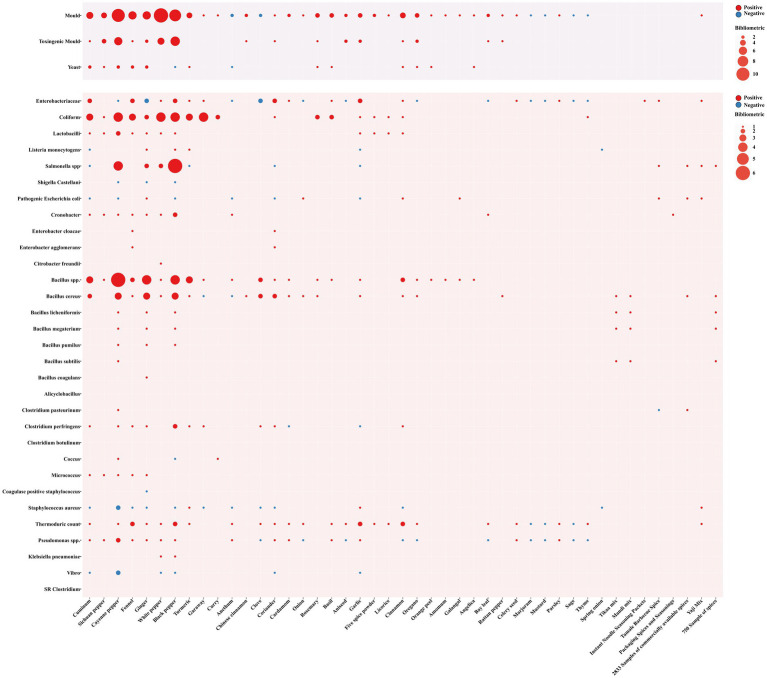
Risk matrix of microbial contamination of spices.

This section presents the microbial contamination status of spiced foods, as elucidated through targeted investigations of key microbial contaminants across a dataset comprising 41 spice varieties (Abdulsalam and Usman, 2019; Banerjee and Sarkar, 2003; Bakobie et al., 2017; Bata-Vidács et al., 2018; Chen et al., 2017; Geeta and Kulkarni, 1987; Garcia et al., 2018; Huang et al., 2015; Koohy-Kamaly-Dehkordy et al., 2013; Lebai Juri et al., 1986; Lu, 2022; Little et al., 2003; Li et al., 2005; Li, 2010; Liu, 2015; Liu et al., 2016; Li et al., 2017; Li et al., 2022; Ma et al., 2015; Motloung et al., 2018; Moore et al., 2019; Oranusi et al., 2013; Rahman et al., 2021; Shi et al., 2003; Sagoo et al., 2009; Shu, 2016; Torpdahl et al., 2023; Witkowska et al., 2011; Wong, 2015; Xue, 2023; Zhang et al., 2007; Zhang, 2009a; Zhang, 2009b; Zhao et al., 2013). Additionally, microculturomics analysis was employed to assess microbial contamination levels in 37 commonly available spices, including cumin, Sichuan pepper, cayenne pepper, and mixed spices, alongside 7 blended spice combinations. Figure 1 illustrates the risk matrix delineating microbial contamination in spices, highlighting the prevalence of fungi and bacteria as prominent contamination risks. However, it is noteworthy that comprehensive reports or studies regarding microbial contamination of numerous spices remain limited or insufficiently documented.

#### 3.1.1 Fungal contamination in food spices

The prevalent fungal contaminants found in spices encompass species such as *Aspergillus* sp., *Penicillium* sp., *Absidia* sp., *Rhizomucor* sp., *Cladosporium* sp., *Mycelia sterilia*, *Alternaria* sp., *Fusarium* spp., and *yeast*. Figure 1 illustrates that spices commonly exhibit elevated levels of fungal contamination (26 out of 33 examined varieties) and pose a diverse spectrum of potential mycotoxin hazards (16 out of 33). Particularly, cumin, capsicum, white pepper, and black pepper exhibited heightened levels of mold pollution and residual mycotoxin risks, including aflatoxin and ochratoxin. Similarly, Sichuan peppercorns, ginger, turmeric, rosemary, basil, star anise, garlic, cinnamon, bay leaves, rattan peppercorns, and oregano demonstrated notable fungal contamination levels. While caraway seed, curry, cardamom, onion, five-spice powder, licorice, orange peel, amomum kernel, grass nut, angelica, celery seed, and parsley also exhibited fungal contamination, comprehensive data regarding mycotoxin presence were lacking. Notably, cloves, dill, marjoram, mustard, sage, and thyme displayed no detectable fungal contamination, attributed in part to the abundant antifungal compounds, such as eugenol, present in cloves (Garcia et al., 2018). Additionally, *yeast* contamination was observed in several spices including cumin, Sichuan pepper, cayenne pepper, ginger, turmeric, rosemary, basil, cinnamon, and oregano, potentially influenced by variations in spice substrate composition.

Fungal contamination typically exhibits intercommunication and a notable resistance to stress factors, facilitating its sustained growth within the low water activity environment characteristic of spices. Garcia et al. conducted an analysis of 200 samples encompassing rosemary, fennel, cinnamon, cloves, black pepper, white pepper, and oregano, leading to the isolation of 51 distinct fungi representing 22 genera. The investigation revealed the prevalence of species such as *Aspergillus ruber*, *Aspergillus chevalieri*, *Aspergillus montevidensis*, and *Aspergillus pseudograviviridis* across multiple spice varieties, alongside *pseudoglaucus*, *Aspergillus penicillioides*, toxin-producing *Aspergillus flavus*, *Aspergillus niger*, and various other fungal species (Garcia et al., 2018).

It is noteworthy that within the scope of the study, the detection rates of *Aspergillus* spp. (32%), *Aspergillus nomius* (12%), and *Aspergillus parasiticus* (4%) were observed in both white pepper and black pepper samples. Additionally, the detection rate of the *Aspergillus niger* complex reached a notable 52%, while *Aspergillus ochraceus* was detected at a rate of 12%. Furthermore, concerning aflatoxin production, 14.2% of isolated strains from black pepper, 66.7% from white pepper, and all strains from white pepper demonstrated the ability to produce aflatoxins. Another investigation conducted by Garcia revealed that 29% of 304 spice samples obtained from vendors in Mexico City tested positive for *Aspergillus niger* and *rhizopus* contamination (Wong, 2015).

Motloung et al. conducted an investigation revealing that 40% of the samples comprising 70 types of South African food spices, including pepper and ginger, were contaminated with aflatoxin, ochratoxin, and other mycotoxins, with 11% of the samples containing a variety of mycotoxins (Motloung et al., 2018). Additionally, in 2014, Chen Yuhang et al. scrutinized spices available in Chengdu, uncovering an average mold count of (8.4 ± 1.5) × 10^3^ CFU/g among 120 samples. Remarkably, 92.5% of these samples tested positive for mycotoxins, with 31.7% exhibiting the presence of aflatoxin B1, ochratoxin A, and fumonisin B (Chen et al., 2017).

#### 3.1.2 Bacterial contamination in food spices

The predominant bacterial contaminants found in spices encompass a diverse array of microorganisms, notably including *Enterobacteriaceae*, *Coliforms, Lactobacilli*, *Cocci*, *Micrococci*, and thermoduric bacteria. Furthermore, spices are susceptible to contamination by *Bacillus* species, *Clostridium perfringens*, *Listeria monocytogenes*, various *Salmonella* serotypes, *Pseudomonas* species, pathogenic *Escherichia coli*, *Cronobacter*, *Enterobacter cloacae*, *Enterobacter agglomerans*, *Citrobacter freundii*, *Klebsiella pneumoniae*, and *Staphylococcus aureus*, among others.

As illustrated in Figure 1, the analysis revealed elevated levels of contamination in various spices by *Enterobacter*, *Coliforms*, *Bacillus*, and *Bacillus cereus* (in this study, statistical analysis was independently conducted for *Bacillus cereus* and other significant contaminants in spices). Subsequently, noteworthy levels of contamination were also observed for *Enterobacter*, *Lactobacillus*, *Cronobacter*, *Clostridium perfringens*, and *Pseudomonas*. Vibrio, typically abundant in aquatic products, displayed minimal contamination levels in spices. However, prevalent foodborne pathogens such as *Listeria monocytogenes*, *Salmonella*, pathogenic *Escherichia coli*, *Staphylococcus aureus*, and *Klebsiella pneumoniae* exhibited high contamination levels in cumin, ginger, black pepper, and white pepper, while data on contamination levels in other spices were either limited or absent. These pathogens demonstrated high detection rates across a broad spectrum of microbial contamination surveys covering various spices. Furthermore, cumin, cayenne pepper, ginger, white pepper, and black pepper generally manifested a diverse array of cross-contamination involving bacteria and fungi. Notably, cross-contamination of *Coliforms*, *Lactobacillus*, *Salmonella*, *Cronobacter*, *Bacillus*, *Bacillus subtilis*, thermoduric bacteria, *Pseudomonas*, and *Clostridium perfringens* was prominently observed in black pepper and white pepper.

Between 1973 and 2010, 71% of the 14 reported food-borne disease outbreaks associated with microbial contaminants were attributed to spices. *Salmonella* was identified as the primary pathogen in 10 cases, while *Bacillus* (comprising 2 cases of *Bacillus cereus*, 2 cases of *Bacillus subtilis*, and 1 case of *Bacillus pumilus*) was determined to be the primary pathogen in the remaining 4 cases (Van Doren et al., 2013). And the U. S. Food and Drug Administration surveyed packaged (dried) spices sold in the U. S., collecting 7,250 retail samples of 11 spice types between November 2013 and March 2015 (Zhang et al., 2017). The study revealed *Salmonella* prevalence estimates of 0.15–0.64% across various spices, including basil leaf, black pepper, coriander seed, curry powder, dehydrated garlic, oregano leaf, paprika, and red pepper (Zhang et al., 2017). Furthermore, within the past decade, the European Union’s Rapid Alert System for Food and Feed (RASFF) has issued over 500 notifications concerning the risk of microbial contamination associated with imported “herbs and spices.” The prevalent microbial contaminants encompass *Salmonella*, *Listeria monocytogenes*, *Bacillus cereus*, pathogenic *Escherichia coli*, and mycotoxins, affecting black pepper, rosemary, ginger, thyme, paprika, parsley, curry, and other spices.

Additionally, in 2022, a substantial number of batches, exceeding 40, were subjected to serious notifications regarding *Salmonella* contamination in black pepper.^2^ Little et al. conducted an analysis of 750 spices and flavorings, revealing the presence of *Bacillus cereus* in 142 (19%) samples, other *Bacillus genera* in 399 (53%) samples, and concentrations of up to 10^4^ CFU/g of *Bacillus cereus* and other *Bacillus* genera in 222 samples (Little et al., 2003). Similarly, Garcia’s investigation identified *Bacillus cereus* in 32 out of 304 spice samples retailed in Mexico City (Garcia et al., 2018). In a separate study by Li et al., the examination of spices sold in Wenzhou unveiled a detection rate of *Cronobacter* in 22.30% (66 strains) of the 296 spices analyzed (Li et al., 2017).

### 3.2 Risk analysis of microbial contaminations in food spices

Based on distinct microbial pollution characteristics, the risks associated with microbial contamination in spices were initially categorized into three groups: pathogenic risk, pathogenic spoilage risk, and spoilage risk, as detailed in Table 1. The identified pathogenic risk groups for spice contamination encompass *Cocci*, *Listeria monocytogenes*, *Shigella*, pathogenic *Escherichia coli*, *Cronobacter*, *Salmonella*, *Klebsiella pneumoniae*, *Staphylococcus aureus*, *Vibrio*, *Clostridium perfringens*, *Botulinum*, *Clostridium perfringens*, *Enterobacter clostridium*, *Citrobacter fredii*, and *Bacillus subtilis*. These pathogens or their associated toxins adhere to spices, subsequently contaminating food products and leading to potential poisoning or gastrointestinal infections upon ingestion.

**Table 1:** Risk classification of microbial contamination in spices.

Risk of disease	Pathogenicity and spoilage risk	Risk of spoilage
*Coccus*	*Toxigenic mold*	*Mold*
*Listeria monocytogens*	*Pseudomonas* spp.	*Yeast*
*Salmonella* spp.	*Enterobacteriaceae*	*Lactobacilli*
*Shigella Castellani*	*Coliform*	*Micrococcus*
Pathogenic *Escherichia coli*	*Bacillus* spp.	*B. licheniformis*
*Cronobacter*	*Campylobacter*	*B. subtilis*
*Staphylococcus aureus*		*Bacillus pumilus*
*Klebsiella pneumoniae*		*Bacillus megaterium*
*Vibro*		*Thermoduric count*
*Clostridium botulinum*		*Bacillus coagulans*
*Clostridium perfringens*		*Alicyclobacillus*
*Enterobacter cloacae*		*Clostridium pasteurinum*
*Enterobacter agglomerans*		
*Citrobacter freundii*		
*Bacillus cereus*		

The microorganisms contributing to the spoilage risk of spices comprise *mold*, *yeast*, *Lactobacillus*, *Micrococcus*, *Bacillus licheniformis*, *Bacillus megaterium*, *Bacillus pumilus*, *B. subtilis*, *Bacillus coagulans*, *Alicyclobacillus*, and *Clostridium pasteurinum*. These spoilage microorganisms can directly result in the deterioration of spices and, upon introduction into food substrates, lead to the spoilage of the entire food products. Furthermore, the microorganisms associated with pathogenic and spoilage risks in spice contamination encompass mycotoxin-producing *molds*, *Pseudomonas*, *coliform*, *Enterobacter group*, *Bacillus*, and *Campylobacter*. These microorganisms not only facilitate the spoilage of spices and food but also present a significant pathogenic risk.

**Figure 2:**
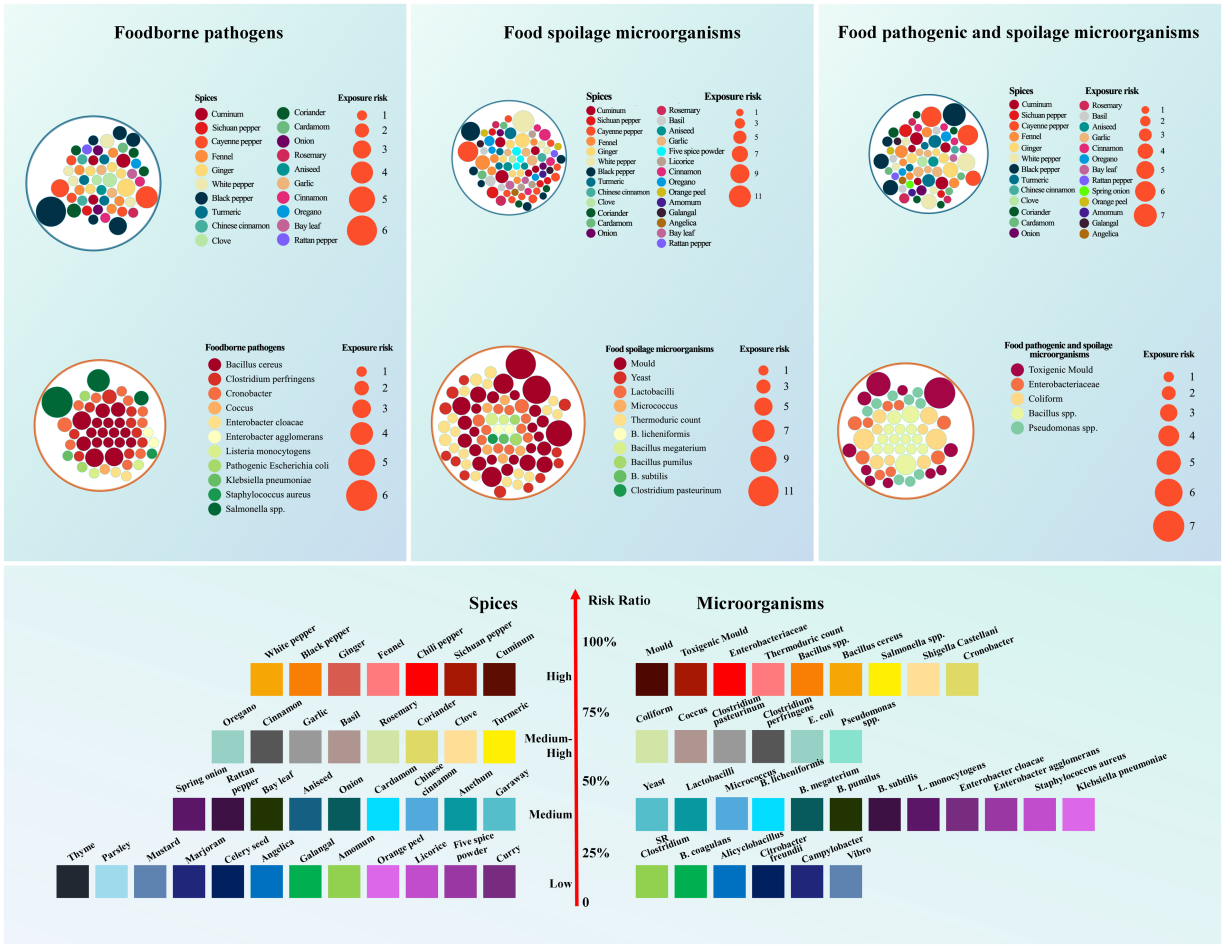
The risk of exposure of spices to microorganisms and the classification of risk levels of spices and microorganisms.

To further evaluate the potential exposure risk of spices to pathogenic microorganisms, spoilage microorganisms, and their combination, a thorough analysis of pertinent data was conducted, and the findings are presented in Figure 2. The examination revealed that cumin, capsicum, black pepper, white pepper, and ginger exhibited a high risk of pathogenic bacteria, spoilage bacteria, and pathogenic spoilage bacteria. These spices demonstrated an elevated likelihood of exposure to specific pathogens such as *Bacillus subtilis*, *Clostridium perfringens*, and *Cronobacter*, while displaying a comparatively lower risk of exposure to *E. coli*, *Klebsiella pneumoniae*, and *Staphylococcus aureus*. Spices exhibited an increased likelihood of exposure to associated spoilage microorganisms, including *molds*, *yeast*, and *thermophiles*, while presenting a lower susceptibility to *Bacillus subtilis*, *Bacillus licheniformis*, *Bacillus megaterium*, *Bacillus parvus pumilus*, and *Clostridium*. Moreover, spices demonstrated a heightened risk of exposure to mycotoxin-producing *molds*, *coliforms*, *Enterobacter*, *Bacillus*, and *Pseudomonas*.

This study undertook a comprehensive assessment of factors including the risk of exposure to microorganisms, prevalent processing methods, consumption patterns, and microbial characteristics. These characteristics encompassed the potential for toxin production, the degree of pathogenicity, inclusion on the Centers for Disease Control and Prevention (CDC) warning list, susceptibility to spoilage, residue risk, and resilience to environmental conditions and storage. Subsequently, risk levels pertaining to spices and their associated microorganisms were determined, scored, and initially categorized, with the outcomes depicted in Figure 2. The classification stratified risk into four tiers: high risk (75–100%), moderate risk (50–75%), moderate risk (25–50%), and low risk (0–25%).

The spices categorized as high-risk encompass cumin, Sichuan pepper, chili pepper, ginger, black pepper, and white pepper. Those deemed medium-high risk include turmeric, cloves, coriander, rosemary, basil, garlic, cinnamon, and oregano. Meanwhile, caraway seed, dill, cardamom, onion, star anise, bay leaf, rattan pepper, and spring onion were identified as medium-risk spices. Spices categorized as low risk comprise curry, five spice powder, licorice, orange peel, amomum, galangal, Angelica, celery seed, marjoram, mustard, parsley, and thyme.

The high-risk microorganisms identified in spices exposure encompass molds, mycotoxigenic *molds*, *Enterobacter*, *thermophilic* bacteria, *Bacillus*, *Bacillus cereus*, *Salmonella*, *Shigella*, and *Cronobacter*. Furthermore, high-risk microorganisms in spices exposure include *coliforms*, *cocci*, *Clostridium*, pathogenic *Escherichia coli*, and *Pseudomonas*. The microorganisms posing a risk in spices exposure consist of *yeast*, *Lactobacillus*, *Micrococcus*, *Clostridium perfringens*, *Bacillus licheniformis*, *Bacillus megaterium*, *Bacillus pumilus*, *Bacillus subtilis*, *Enterobacter cloacae*, *Listeria monocytogenes*, *Enterobacter agglomerans*, *Staphylococcus aureus*, and *Klebsiella pneumoniae*. Lastly, the low-risk microorganisms in spices exposure include *Clostridium enterica*, *Bacillus coagulans*, *Bacillus aliphaticacid*, *Citrobacter freundii*, and *Vibrio*.

### 3.3 Early warning framework for microorganism in food spices

**Figure 3:**
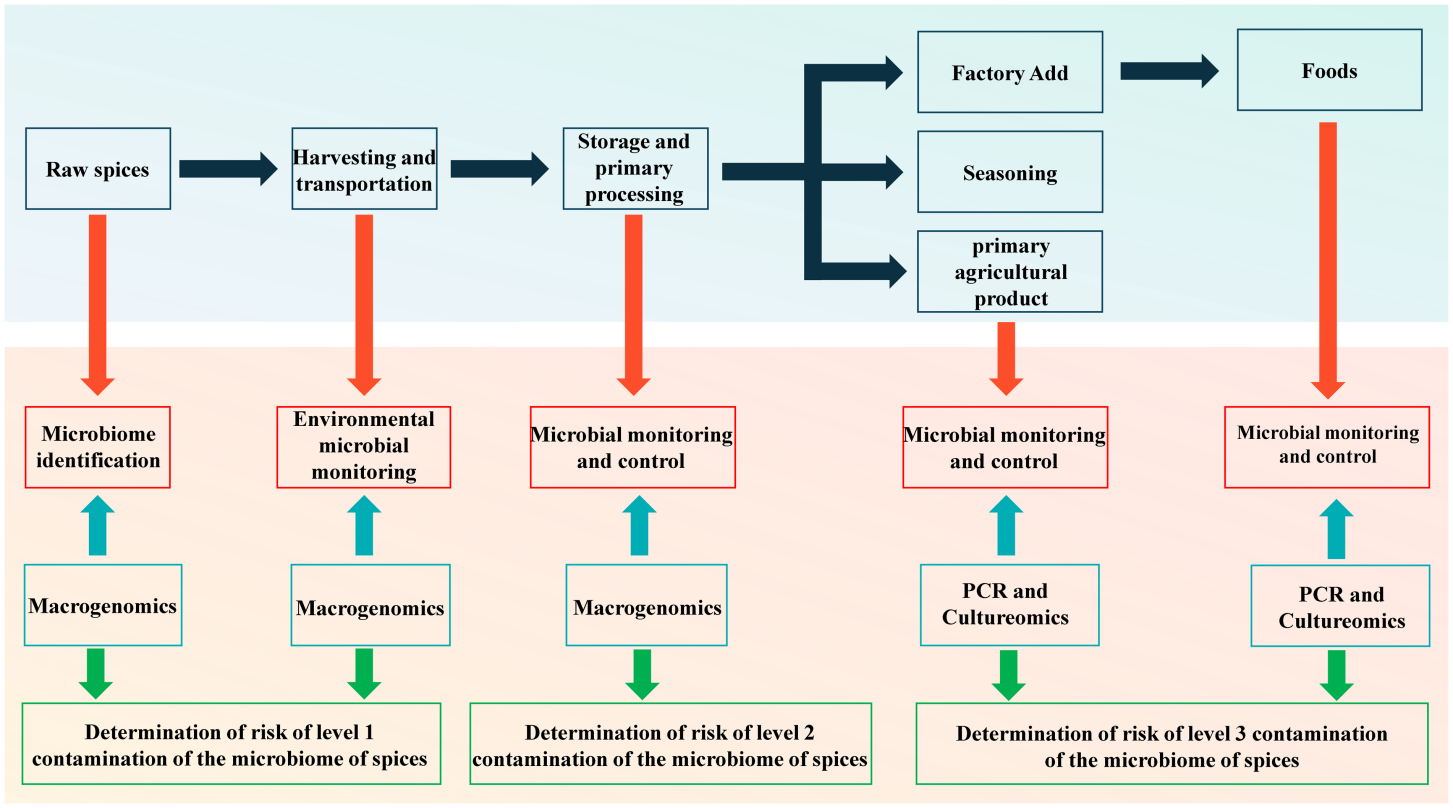
Early warning framework for microorganism import of spices.

Monitoring the levels of microbial contaminants present in spices to identify potential biohazards plays a crucial role in providing essential toxicological data for the safe use of spices, enhancing the accuracy of dietary risk assessment, and implementing effective control measures. In light of this, we have developed an early warning framework for the importation of microorganisms in food spices, outlined in Figure 3. The sources of microbial contamination in spices primarily stem from cross-contamination occurring during various stages in the supply chain, including soil environments pre-harvest, transportation, and storage, characterized by extensive diversity and complexity. As such, our framework emphasizes qualitative or semi-quantitative analysis of the predominant microflora present in spices, rather than solely focusing on quantifying specific spoilage and pathogenic microorganisms, as is commonly practiced. This approach aims to provide a deeper understanding of the risks associated with microbial importation into spices.

Given that spices are inherently host to a diverse array of bacteria and fungi, the on-going advancements in metagenomics and sequencing technologies present an opportunity for a comprehensive exploration of the microbiome within spices. Metagenomic sequencing facilitates both qualitative and quantitative analysis of the microbiome, there-by addressing the constraints associated with the culturability of microorganisms in traditional culturomics. Additionally, this technology enables the collection of comparative microbiome data across various spices in different spatio-temporal contexts, thereby establishing an ever-expanding dataset of spice microbiomes. These insights are invaluable for conducting risk assessments related to microbial exposure in spices and serve as a fundamental basis for devising microbial control strategies spanning the entire spectrum of spice production, from cultivation to consumption.

Given the potential association between microbial contamination of spices and factors such as their soil environment, storage, processing methods, and eventual use (including incorporation into processed foods in manufacturing facilities or direct consumption), the establishment of an early warning framework for microbial importation can effectively delineate level 1, 2, and 3 contamination risks associated with the spice micro-biome. This framework holds significant promise in offering valuable insights for the sustainable management of agricultural production processes involved in spice cultivation and harvesting. Furthermore, it can serve as a crucial guide for devising monitoring programs and implementing control measures at critical junctures within the processing, storage, and utilization of spices. Simultaneously, it serves as a pivotal reference for the development of quality and safety traceability systems for spices and the formulation of national standards governing spice quality and safety (Wang and Wang, 2021).

## 4 Conclusion

The widespread presence of pathogenic and/or spoilage microorganisms in spices can not only lead to the spices’ own deterioration but also compromise food quality and sensory dimensions when incorporated into food products, thereby heightening the risk of spoilage and foodborne illnesses. This investigation encompassed 41 datasets pertaining to microbial contamination in spices, elucidated prevalent levels of microbial contamination observed in spices, evaluated the associated exposure risks, and delineated the significant pathogenic, spoilage, and pathogenic-spoilage microorganisms, subsequently categorizing the associated risk levels. *Salmonella*, pathogenic *Escherichia coli*, *Coliforms*, *Clostridium perfringens*, *Cronobacter*, *Bacillus cereus*, *Bacillus* spp., *molds*, and mycotoxin-producing *molds* were identified as high-risk contaminants in spices. Specifically, cumin, Zanthoxylum, cayenne pepper, ginger, black pepper, and white pepper were classified as high-risk spices, warranting specialized monitoring of their microbial contamination levels during usage. Notably, numerous spices have received inadequate attention in terms of food safety and public health considerations thus far. To address this, we proposed an early warning framework for identifying microbial input in spices. Leveraging sequencing technology, an ever-expanding dataset of spice microbiomes can be established, providing pivotal data for assessing the microbial exposure risks associated with spices and serving as a fundamental basis for devising comprehensive microbial control strategies across various stages of the spice supply chain, from cultivation to consumption.

## Data Availability

The original contributions presented in the study are included in the article/supplementary material, further inquiries can be directed to the corresponding author.

## Footnotes

^1^ https://www.Chiplot.online

^2^ https://webgate.ec.europa.eu/rasff-window/screen/search
